# Charting the champions: distinguishing the career paths of international futsal champions – a retrospective study

**DOI:** 10.5114/biolsport.2026.161098

**Published:** 2026-07-27

**Authors:** Diogo Mendes, Diogo V. Martinho, Bruno Travassos, Élvio R. Gouveia, Nestor Ordoñez Saavedra, Pedro Campos, Liliana C. Baptista, Hugo Sarmento

**Affiliations:** 1 Universidade de Coimbra, CIPER, Faculdade de Ciências do Desporto e Educação Física, Coimbra, Portugal; 2 CIPER, Faculdade de Motricidade Humana, Universidade de Lisboa, Cruz-Quebrada-Dafundo, Portugal; 3 Research Center in Sports Sciences, Health Sciences and Human Development (CIDESD), Department of Sport Sciences, Universidade da Beira Interior, Covilhã, Portugal; 4 Portugal Football School, Portuguese Football Federation, Portugal; 5 Department of Physical Education and Sport, University of Madeira, Funchal, Portugal; 6 Laboratory of Robotics and Engineering Systems, Interactive Technologies Institute, Funchal, Portugal; 7 Universidad de Ciencias Aplicadas y Ambientales (UDCA), Bogotá, Colombia; 8 Department of Informatics Engineering and Interactive Media Design, University of Madeira, Funchal, Portugal; 9 WoWSystems Informática Lda, Funchal, Portugal

**Keywords:** Expert, Coach-led practice, Early diversification, Talent pathways

## Abstract

Understanding the activities that futsal players engage in during their developmental years is essential for grasping the attainment of expertise. Most studies have been conducted in soccer, and there is a lack of research specific to futsal. Given the investment of the Portuguese Football Federation in futsal, exploring the developmental pathways leading to elite levels could provide significant insights for optimizing the long-term athletic development of futsal players. The present study describes the developmental activities of elite players (those who have won the European or World Championship) and national players (who have played in the 1^st^ Portuguese Division but have never attained international level status). The sample comprised 30 national players and 19 elite players. Questionnaires were individually applied to each player. The analysis included the variables related to each player’s developmental trajectory. Elite players initiated coach-led futsal practice later than national-level players (*t* = 2.374, *p* = 0.022, d = 0.696), with similar trends observed for the initiation of competitive futsal (*t* = 1.040, *p* = 0.058, d = 0.569) and peer-led practice in other sports (*t* = 2.076, *p* = 0.061, d = 0.995). Although age of specialization was higher in elite players (13.3 ± 4.1 vs. 11.3 ± 4.0 years), no significant differences were observed between groups (*t* = 1.663, *p* = 0.113, d = 0.473). Across the developmental pathway, elite players accumulated greater coach-led futsal practice volume (*t* = 4.343, *p* < 0.001, d = 1.273), while no differences were found for peer-led practice, participation in other sports, or competition exposure. Developmental trajectories showed a progressive increase in coach-led futsal practice in both groups, with significant between-group differences only observed in adulthood (≥ 23 years: *t* = 3.251, *p* = 0.003, d = 1.170). These findings may inform the Portuguese Football Federation by suggesting that later specialization and sustained engagement in coach-led futsal and other sports may be relevant for the development of elite players.

## INTRODUCTION

The processes of identifying and developing athletes from an early age into elite competitors are influenced by multiple constraints, highlighting the need for a multidimensional perspective in the study of talent [1, 2]. In recent years, there has been an increase in investment by public organizations and academies aimed at enhancing the understanding of athlete identification and development [3, 4]. Talent identification is defined as recognizing young participants who have the potential to become elite athletes, while talent development refers to the ongoing process of providing environmental support to accelerate their long-term potential [5, 6]. However, identifying potential elite athletes is fraught with challenges, such as the difficulty of predicting the future and the constantly changing needs of sports [7]. Consequently, there has been considerable attention directed toward investigating developmental activities that can enhance athlete readiness [8–10]. Understanding the types and number of practices athletes experience during their developmental years is central to the attainment of expertise [11–13]. This research focuses on the developmental activities of Portuguese male futsal players.

The analysis of developmental pathways concerning the amount and type of practices has primarily focused on male and female soccer players, as well as Olympic participants [8, 9, 13, 14]. For instance, players who reached the National Team in Germany engaged more in non-soccer activities compared to amateur players [14]. Additionally, Olympic/World Championships medalists reported greater involvement in various sports, accumulating more practice hours from ages 10 to 18 than those who did not medal [13]. Among female soccer players, professionals began participating in soccer and receiving coaching from adults at a significantly earlier age than ex-academy players, indicating the importance of early training [8]. The differing results across studies can be explained by the talent development systems implemented within each sport organization. These systems, in turn, are influenced by socio-cultural and historical constraints [15]. Therefore, examining the developmental trajectories of futsal players who have won European or World Championships, in contrast to those who have never attained the national team, may provide insights into how the quantity and type of practice impacts expertise in the sport [6]. Generalizations from studies conducted in soccer or other sports should be interpreted with caution when applied to futsal, given its unique structural and task constraints [16]. Futsal is characterised by higher tactical density, frequent player rotations, reduced playing space, and increased technical involvement per player, which likely shape distinct developmental pathways in terms of practice quantity and type [17, 18].

The popularity of futsal has increased exponentially, particularly following the UEFA Futsal EURO 2022, which attracted 19.8 million viewers [19]. Participation rates in futsal in Portugal have also risen over the years. For instance, the number of registered male participants in the Portuguese Football Federation increased from 24.181 in 2011 to 36.543 in 2023 [20]. Consequently, it is not surprising that research on futsal has received considerable attention, particularly regarding its physical, technical, and tactical demands [21–25]. However, studies focusing on the developmental activities of futsal players remain limited. One study, based on semistructured interviews with 23 futsal players from various countries (Brazil, Spain, Italy, Portugal, and Argentina), concluded that coaches and families play significant roles in the success of these athletes, while unstructured play is recognized as a vital practice for achieving expertise [26]. Additionally, another study involving elite Portuguese futsal players found that these athletes tend to be committed to futsal from an early age [27]. However, this research did not explore the extent of their involvement in other sports or whether their practice was primarily led by a coach or peers.

Understanding the pathways to outstanding performance levels is crucial for governing bodies and practitioners seeking to comprehend the processes leading to success in international competitions. In Portugal, the developmental model for futsal practice has undergone adjustments since 1999 [28], coinciding with the recent success of the senior national team in international tournaments. However, research on developmental pathways in futsal remains limited, and a broader understanding of the practices of successful players is needed to inform the Portuguese Football Federation in refining its current model. Previous studies have primarily focused on soccer or elite athletes participating in Olympic or World Championships [29, 30], and it remains unclear whether their findings are transferable to futsal, given its unique structural and task constraints. Therefore, examining developmental pathways in futsal may provide important insights into the context-specific nature of talent development. Given the increasing global popularity of futsal, this study aimed to describe and compare the developmental activities of players across competitive levels, focusing on the timing, volume, and type of practice.

## MATERIALS AND METHODS

### Participants and ethics approval

The study included 49 Portuguese futsal players categorized into two distinct groups based on the competitive level: (1) national-level players (n = 30), competing at the national first Portuguese league but never represented the national team; (2) elite-level players (n = 19) who were futsal athletes who won senior World and/or European competitions, representing national Team. The current project is supported by the Portuguese Football Federation through the Portuguese Football School. One of the authors, BT, serves as the main physiologist for the adult national team, which facilitates the selection and recruitment of elite players. To recruit participants, club representatives of players competing in the highest tier of futsal in Portugal were contacted individually via email. The contact information was provided by the Portuguese Football Federation and performed by the first author of the manuscript. The sample was selected using a convenient sampling method. Data was collected during 2022–2023 and 2023–2024 competitive seasons. After the purpose of the study was explained, all the players who were initially contacted agreed to participate. The study was approved by the Ethics Committee of the Faculty of Sport Sciences and Physical Education, University of Coimbra (approval code: CE/FCDEF-UC/00842021; approval date: 25 March 2022).

### Survey

Futsal players completed a structured survey comprising three sections: (1) demographic and contextual information, including athletes’ background characteristics; (2) training practices in futsal, focusing on training experiences and session characteristics; and (3) involvement in sport, assessing participation in futsal and other sports across development. Retrospective questionnaires have been shown to be reliable and valid methods for characterizing athletic participation from childhood to adulthood [14, 31–33] and have been widely applied to examine athletes’ developmental trajectories across different sport contexts [14, 31–34]. The following variables were extracted for analysis: age at initiation of peer-led and coach-led practice in futsal; age at initiation of competition in futsal; age of specialization in futsal; age at initiation of peer-led and coach-led practice in other sports; age at initiation of competition in other sports; volume of practice in coachled futsal and other sports, as well as peer-led practice in other sports; and competition exposure, expressed as minutes played per season and number of matches per season in futsal. Additionally, within coachled futsal practice, the proportion of time dedicated to technical and tactical exercises, physical conditioning, and specialized practice (i.e., training activities tailored to players’ individual physical, technical, and tactical characteristics) was assessed.

### Data collection

Before administering the questionnaire, the first author contacted each player via phone or email to explain the purpose of the questions and to schedule an appointment for the assessment. Players completed the questionnaire in person under the supervision of the first author, who provided instructions and clarifications regarding the practice activities. A comfortable and confidential environment was arranged for the players to fill out the questionnaire, which took, on average, 60 to 90 minutes to complete.

### Statistical analysis

Descriptive statistics (range, mean, standard deviation, and 95% confidence intervals for the mean) were calculated for national- and elite-level groups across the following variables: chronovariables (age at initiation of peer-led and coach-led practice in futsal; age at initiation of competition in futsal; age of specialization in futsal; age at initiation of peer-led and coach-led practice in other sports; age at initiation of competition in other sports); practice volume (coach-led practice in futsal and other sports, as well as peer-led practice in other sports, expressed as the average across the developmental trajectory and by age group); and competition exposure (expressed as minutes played and number of matches per season in futsal). Between-group differences were assessed using independent samples t-tests. Effect sizes were calculated using Cohen’s d and interpreted as follows [35]: d < 0.2 (trivial), 0.2 ≤ d < 0.6 (small), 0.6 ≤ d < 1.2 (moderate), 1.2 ≤ d < 2.0 (large), 2.0 ≤ d < 4.0 (very large), and d ≥ 4.0 (nearly perfect). The microstructure of practice (expressed as the percentage of time devoted to technical/tactical activities, physical conditioning, and specialized practice) was analysed across the season (pre-season vs. in-season) for both groups. The significance level was set at *p* < 0.05, and all statistical tests were two-tailed. Statistical analyses were performed using SPSS (version 28.0; IBM Corp., Armonk, NY, USA) and GraphPad Prism (version 5.00; GraphPad Software, San Diego, CA, USA).

## RESULTS

Descriptive statistics of chronovariables related to futsal and participation in other sports, including practice volume (hours per year) in coach-led and peer-led activities, as well as competition exposure (minutes and matches per season), across the developmental pathway (from 6 to 23 years) of national and international players are presented in Table 1. On average, elite players initiated coach-led practice, competitive participation, and specialization in futsal later than national-level players. Regarding participation in other sports in coach-led settings, although substantial variability was observed among elite players, both groups tended to initiate such practice during late adolescence. Across the developmental pathway, elite players accumulated greater annual practice volume in futsal (coachled) as well as in other sports (both coach- and peer-led). In contrast, competition exposure, in terms of time and match frequency, was comparable between groups.

**TABLE 1:** Descriptive statistics for chronovariables, annual training volume, and matches players in futsal players of contrasting competitive levels.

Variable	Descriptive statistics by competitive level					

	national			elite		

	range	mean (95% CI)	standard deviation	range	mean (95% CI)	standard deviation
**Chronovariables – futsal**						
age at initiation peer-led practice, yrs	(4; 21)	11.6 (9.7 to 13.4)	4.4	(6; 18)	11.2 (9.0 to 13.7)	4.1
age at initiation coach-led practice, yrs	(3; 16)	8.6 (7.3 to 10.0)	3.6	(5; 18)	11.2 (9.3 to 13.2)	4.1
age at initiation competition, yrs	(6; 16)	10.2 (9.1 to 11.3)	3.1	(5; 18)	12.1 (10.3 to 13.8)	3.7
age of specialization, yrs	(3; 17)	11.3 (9.8 to 12.8)	4.0	(6; 18)	13.3 (11.2 to 15.3)	4.1

**Chronovariables – other sports**						
age at initiation peer-led practice, yrs	(10; 23)	16.1 (13.7 to 18.6)	4.0	(8; 39)	20.7 (10.2 to 31.2)	11.3
age at initiation coach-led practice, yrs	(5; 16)	7.9 (4.4 to 11.3)	3.8	(6; 16)	8.8 (6.9 to 10.8)	3.2
age at initiation competition, yrs	(7; 17)	10.5 (8.4 to 12.7)	3.5	(8; 16)	10.1 (7.4 to 12.9)	3.0

**Volume of practice, hrs**						
coach-led practice futsal	(128; 391)	220 (198 to 241)	57	(193; 450)	304 (193 to 450)	80
peer-led practice futsal	(31; 753)	183 (114 to 251)	176	(24; 698)	185 (98 to 272)	181
coach-led practice other sports	(18; 180)	100 (81 to 118)	44	(36; 188)	114 (83 to 144)	53
peer-led other sports	(4; 48)	17 (11 to 23)	13	(3; 168)	31 (10 to 63)	48

**Games played – futsal**						
min · season−1	(126; 1064)	526 (430 to 622)	255	(210; 1298)	491 (378 to 605)	236
matches · season−1	(10; 40)	20 (11 to 40)	8	(11; 40)	20 (16 to 24)	8

95% CI (95% confidence intervals).

Between-group comparisons revealed a statistically significant difference in the age at initiation of coach-led futsal practice (*t* = 2.374, *p* = 0.022, d = 0.696). The age at initiation of competitive futsal (*t* = 1.040, *p* = 0.058, d = 0.569) and the age at initiation of peer-led practice in other sports (*t* = 2.076, *p* = 0.061, d = 0.995) showed trends towards statistical significance. Additionally, the age of specialization was higher in elite players (13.3 ± 4.1 years) compared to national-level players (11.3 ± 4.0 years); however, no statistically significant differences were observed between groups (*t* = 1.663, *p* = 0.113, d = 0.473) (Figure 1).

**FIG. 1:**
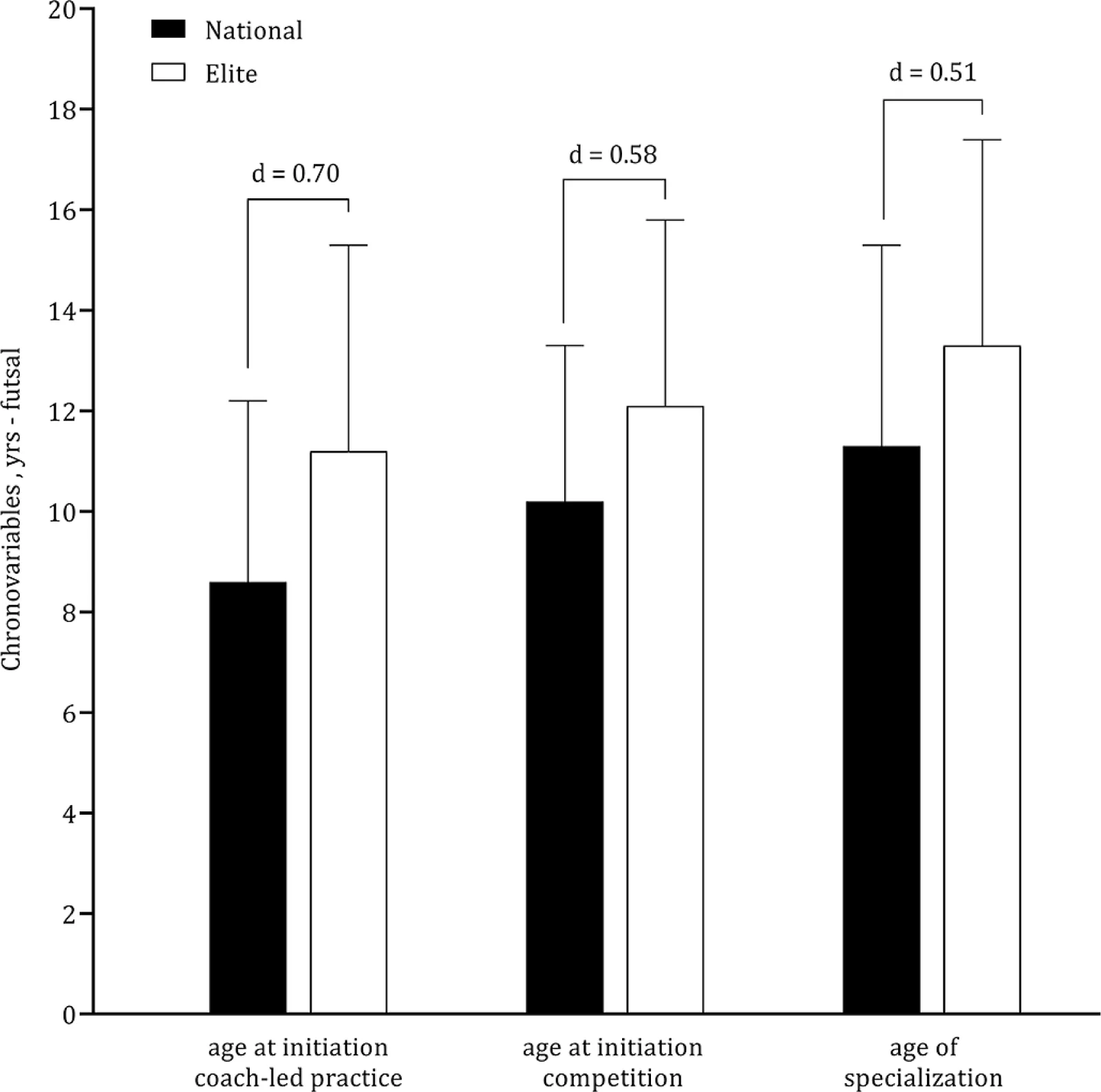
Comparison between elite and national-level players in coach-led futsal practice (panel A), age at initiation of competition (panel B), and age of specialization (panel C). Effect sizes are presented as Cohen’s d.

Across the developmental years, elite players accumulated significantly more time in coach-led futsal practice than national-level players (*t* = 4.343, *p* < 0.001, d = 1.273). No significant betweengroup differences were observed for the remaining variables, including peer-led futsal practice, coach-led practice in other sports, peerled practice in other sports, and number of games played.

Analysis of developmental trajectories indicated a progressive increase in coach-led futsal practice across age categories in both groups. Overall, elite players consistently accumulated more time in coachled futsal practice than national-level players (≤ 11 years: elite = 150 ± 71, national = 99 ± 62; 12–14 years: elite = 196 ± 54, national = 177 ± 81; 15–18 years: elite = 241 ± 98, national = 245 ± 81; 19–22 years: elite = 358 ± 126, national = 341 ± 104; ≥ 23 years: elite = 461 ± 124, national = 327 ± 95). However, between-group differences were only statistically significant and large in the ≥ 23 years category (t = 3.251, p = 0.003, d = 1.170). Although elite players accumulated, on average, greater involvement in coach-led practice in other sports during the developmental years, no statistically significant differences were observed between groups (Figure 2). No between-group differences were observed in peer-led practice in other sports; however, substantial inter-individual variability in practice volume was evident in both groups.

**FIG. 2:**
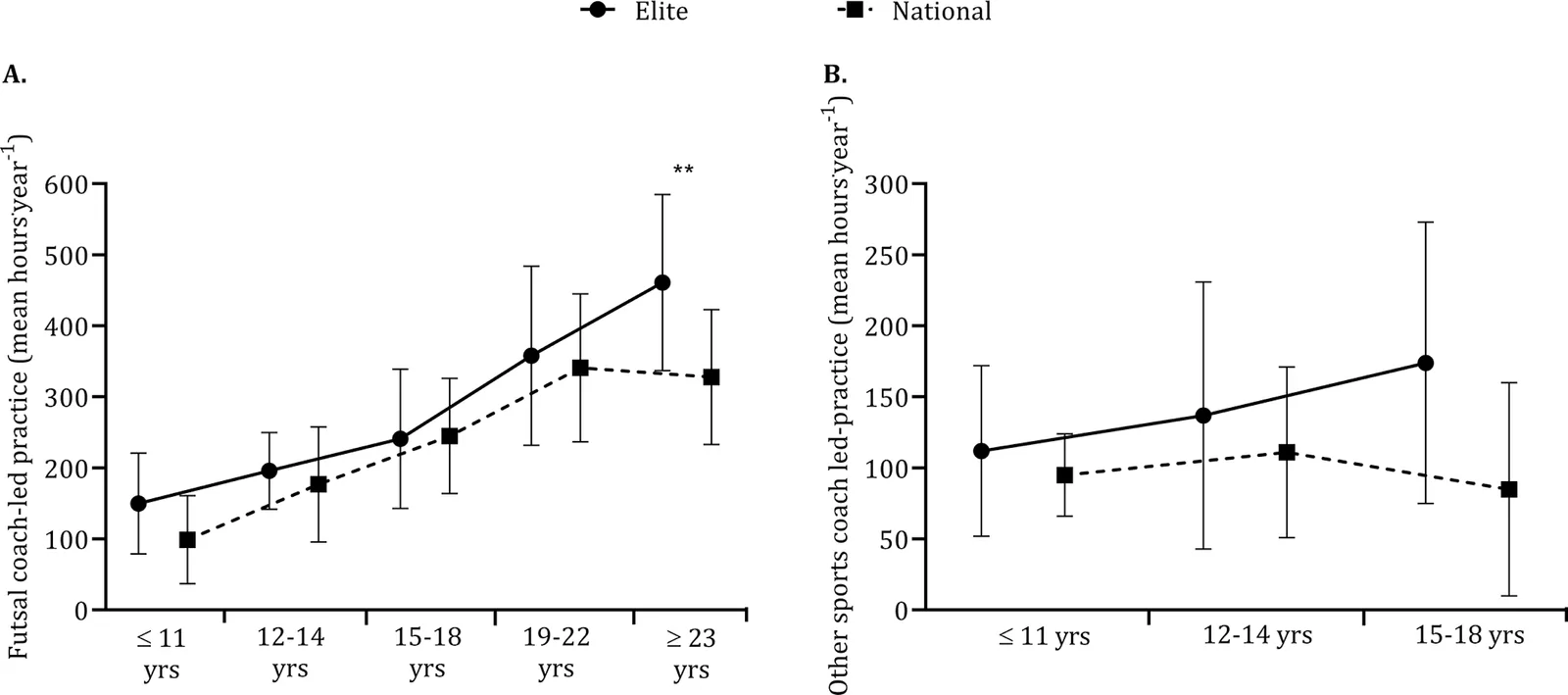
Coach-led practice volume in futsal (panel A) and other sports (panel B) in elite and national-level players across the developmental years. ** p < 0.01

Figure 3 illustrates the percentage of time dedicated to technical/tactical activities, physical conditioning, and specialized practice throughout athletic development by period of the season (panel A: pre-season; panel B: in-season). During the pre-season, elite players showed a progressive reduction in the proportion of technical/tactical training over time, accompanied by increases in physical conditioning and specialized practice. In national-level players, a reduction in technical/tactical activities and a marked increase in physical conditioning were observed from childhood to 22 years of age, whereas the proportion of time allocated to specialized practice remained relatively stable from approximately 13 years onwards.

**FIG. 3:**
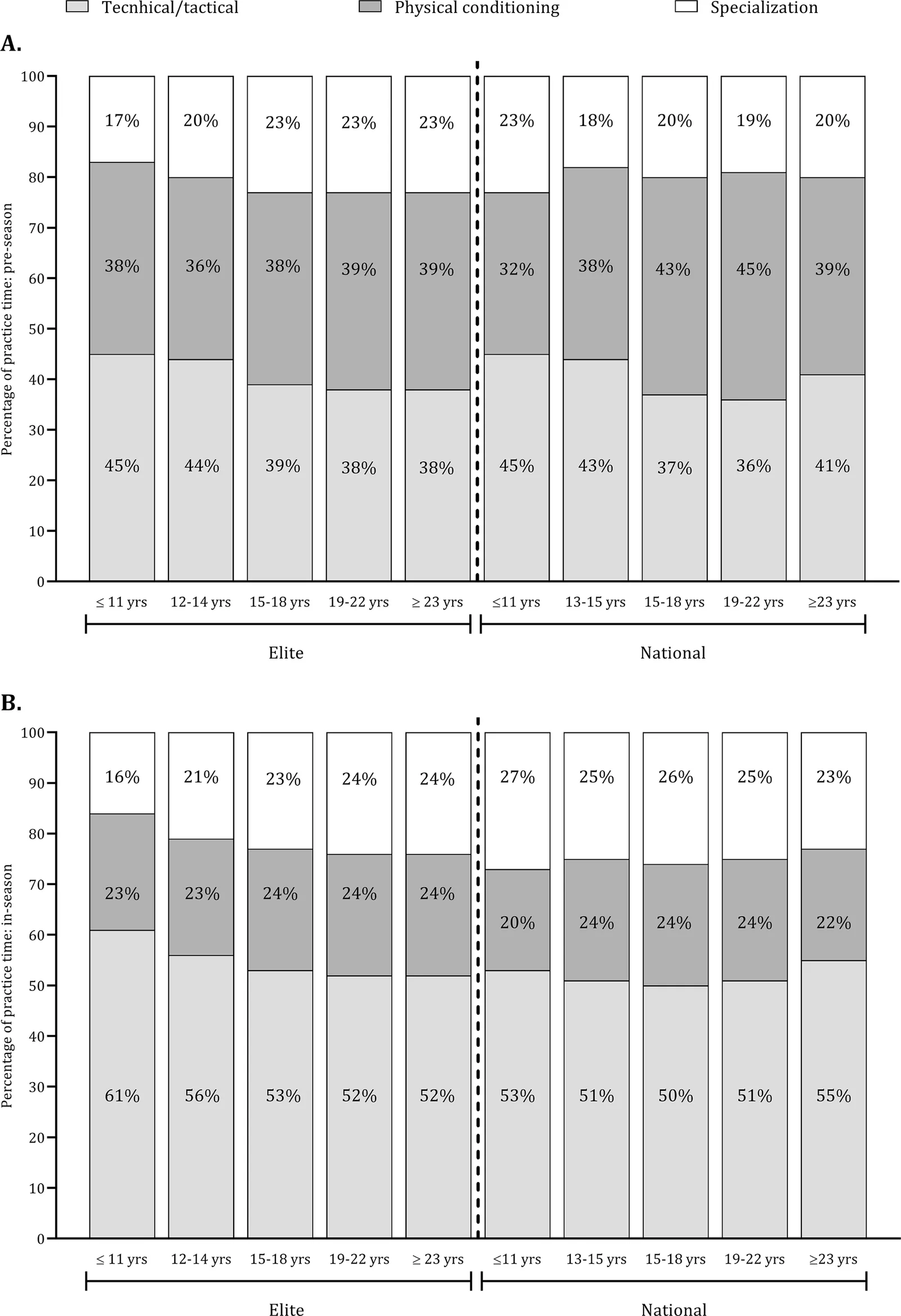
Percentage of time devoted to technical/tactical activities, physical conditioning, and specialized practice in elite and national-level players during the pre-season and in-season across the developmental years.

During the in-season period, elite players reduced the proportion of technical/tactical activities from childhood until approximately 15 years of age, after which it remained relatively stable. Specialized practice increased until mid-adolescence, while the proportion of physical conditioning remained relatively constant across the developmental trajectory. In contrast, national-level players exhibited relatively stable distributions of technical/tactical activities, physical conditioning, and specialized practice across development, with technical activities accounting for approximately 50% of total training time and similar proportions allocated to physical and specialized components.

Table 2 presents the number of other sports in which futsal players participated, categorized by competitive level and distinguishing between coach-led and peer-led activities. National-level players participated in nine different sports in coach-led settings, whereas elite players engaged in twelve different sports. Peer-led practices were observed in both groups. Notably, one elite player reported participation in two coach-led sports (i.e., padel and kickboxing). Additionally, fifteen national-level players and nine elite players reported ongoing participation in various peer-led activities.

**TABLE 2:** The type of sport engaged in, and the number of players by competitive lever and type of practices that engaged.

Type of sport	Coach-led practices of other sports		Peer-led practices of other sports	

	Number of players		Number of players	

	Competitive level		Competitive level	

	National	Elite	National	Elite
Football	13	8	11	1
Swimming	12	2	0	1
Athletics	3	1	0	0
Tennis	2	2	8	2
Table tennis	3	3	1	0
Volleyball	2	0	2	0
Surf	1	1	0	0
Gymnastics	1	0	0	0
Basketball	1	0	3	0
Handball	0	3	0	0
Rugby	0	1	0	0
Karate	0	2	0	0
Kickboxing	0	1	0	0
Boxing	0	1	0	0
Padel	0	1	8	6
Canoeing	0	0	1	0
Badminton	0	0	1	0
Spikeball	0	0	1	0
Running	0	0	2	1
Cycling	0	0	1	1
Beach football	0	0	2	2
Body board	0	0	1	0
Never practiced any coach led-sport	6	5		
Never practiced any peer-led activities			9	7

## DISCUSSION

In this retrospective study, we compared the developmental pathways of male adult Portuguese futsal players who achieved national team status and won European or World Championships with those competing in the Portuguese first division who had not been selected for the national team. The findings provide insights into the developmental pathways associated with expertise in futsal: (1) elite players initiated coach-led futsal practice at a later age than national-level players; (2) the age of initiation of competition and specialization in futsal tended to occur later in elite players; (3) elite players accumulated greater practice volume in both coach-led and peer-led activities, in futsal as well as in other sports; (4) differences in the distribution of practice activities were observed between groups across development; and (5) both groups reported engagement in coach-led and peer-led practice contexts.

The present study provides further insight into the attainment of expertise in futsal while complementing recent research on the likelihood of reaching the international adult level in sport. The emergence of national teams represents an important milestone in understanding the developmental pathways of futsal players [28]. Following the establishment of youth national futsal teams, evidence suggests that both the total number of seasons played and the number of seasons spent in the 1^st^ Division are relevant factors in achieving international adult status [28]. In this context, players selected for the adult national team were classified as international, whereas in the present sample, international-level players were defined as those who have won European or World Championships. However, Mendes et al. [28] did not examine the type or amount of practice in futsal or in other sports. The present findings indicate that elite players tend to initiate training, competition, and specialization later than those who do not reach the international level. This pattern is consistent with previous research in other sports. Specifically, players competing in national teams and the Bundesliga tend to specialize in soccer approximately two years later than amateur players [14], while Olympic and World Championship medalists have been shown to begin practicing their main sport later than nonmedalists [13]. Moreover, elite players reported greater involvement in other sports, particularly in coach-led contexts, as well as higher practice volume in these activities during the developmental years. Collectively, these findings suggest that later specialization and engagement in multiple sports may be associated with the attainment of elite performance in futsal [36].

The Developmental Model of Sport Participation is frequently used to explain the attainment of expertise in sport. This model comprises three phases [36, 37]: the sampling years (6–12 years), the specializing years (13–15 years), and the investment years (16 years and older). During the sampling years, athletes are encouraged to engage primarily in deliberate play activities. The specializing years are characterised by a combination of deliberate play and deliberate practice, representing a transition phase before committing to a single sport during the investment years. In the present study, elite players reported specializing in futsal at approximately 13 years of age, while also engaging in coach-led practice and competitive participation in other sports from early developmental stages (approximately 9 and 10 years of age, respectively). These findings may suggest that elite futsal players are exposed to a variety of structured sporting activities, rather than exclusively deliberate play, during the sampling years, and tend to specialize during early adolescence, broadly aligning with the Developmental Model of Sport Participation. However, the greater involvement in structured activities during the sampling years may indicate some deviations from the model.

Interestingly, the volume of coach-led practice in both futsal and other sports was consistently higher across the developmental pathway in elite players compared to national-level players. These findings indicate that, although elite players tend to start and specialize later in futsal, they accumulate greater amounts of coach-led practice, particularly during late adolescence and adulthood. Engagement in multiple sports has been suggested to contribute to the development of expertise [38, 39], and the present study shows that the annual volume of coach-led practice in both futsal and other sports differs between competitive levels. A meta-analysis involving 9,241 athletes across multiple sports and countries reported that practice in the main sport is associated with success in youth athletes, whereas peer-led practice does not significantly predict performance in either adult or junior athletes [39]. In the present study, coach-led practice in futsal and other sports appears to be more prominent among elite players, whereas peer-led practice showed substantial inter-individual variability and did not clearly distinguish between competitive levels. These findings differ from those reported in the aforementioned meta-analysis but are partially aligned with a recent cross-sectional study of female soccer players, in which professional athletes accumulated significantly more hours of practice during childhood (+884 hours), early adolescence (+134 hours), and late adolescence (+151 hours) compared to ex-academy players [8]. While previous reviews have combined data across multiple sports and countries [38, 39], the present study focuses on a specific sport (futsal) and competitive context, providing a more context-specific understanding of developmental pathways.

The observed differences in practice volume across studies may be explained by the talent development models prevalent in each country, which are shaped by various constraints, including sociocultural and economic factors [1, 15]. For instance, a survey conducted among 25 adolescent soccer players from a small island indicated greater engagement in peer-led activities than in coach-led practice, largely due to the limited availability of qualified coaching and resources [40]. In the Portuguese context, the substantial increase in investment and structural organization of futsal promoted by the Portuguese Football Federation may help explain the greater prominence of coach-led practice in the development of elite players. These findings suggest that the dichotomy between early specialization and early diversification may not fully capture the complexity of pathways leading to expertise in futsal [41]. In this regard, elite players appear to combine engagement in multiple sports with higher volumes of coach-led practice compared to their national-level peers.

The microstructures of training sessions during developmental have been examined in elite athletes [14, 30, 42]. The investigation of microstructure training practices complements the analysis of macrostructure [43]. Firstly, the microstructure of practice over the developmental years various according to the period of the season. Secondly, the current results shows that elite players progressively adjust the content of training sessions across development and seasonal phases, potentially reflecting a more structured and periodized approach to training. In contrast, the more stable distribution observed in national-level players may indicate less variation in training stimuli across developmental stages.

The present study has several limitations that should be acknowledged. First, the sample was recruited using a convenience sampling method and was limited to a single country, which may introduce selection bias and restrict the generalizability of the findings. Second, the sample size of the national-level group (i.e., players competing in the 1^st^ Division) may not be fully representative of the broader population. Nevertheless, the study provides a unique opportunity to include elite players who have won European and World Championships. The overall sample size (n = 49) is comparable to, or larger than, previous studies examining developmental trajectories in elite athletes or soccer players [8, 10, 29, 30, 44, 45]. Third, the present study did not account for the specific constraints involved in the design of futsal training sessions. Finally, although validated and reliable questionnaires were used to assess developmental activities [8, 14, 32, 33], retrospective recall methods are inherently subject to memory bias, which may have influenced variability within and between groups.

## CONCLUSIONS

Elite futsal players tend to specialize later in their careers and are often engaged in multiple sports. Greater involvement in coach-led practice appears to be associated with the attainment of expertise. These findings may inform those involved in talent development within the Portuguese Football Federation by highlighting the potential relevance of combining futsal practice with participation in different sports. Future research should further explore how players perceive and evaluate their developmental activities, as well as identify the most appropriate training practices implemented by elite coaches to support long-term athlete development in futsal.
